# Genetic depletion does not prevent rapid evolution in island‐introduced lizards

**DOI:** 10.1002/ece3.10721

**Published:** 2023-11-27

**Authors:** Stéphanie Sherpa, Josephine R. Paris, Iolanda Silva‐Rocha, Viola Di Canio, Miguel Angel Carretero, Gentile Francesco Ficetola, Daniele Salvi

**Affiliations:** ^1^ Dipartimento di Scienze e Politiche Ambientali Università degli Studi di Milano Milano Italy; ^2^ Dipartimento di Medicina Clinica, Sanità Pubblica, Scienze della Vita e dell'Ambiente Università degli Studi dell'Aquila L'Aquila‐Coppito Italy; ^3^ Centro de Investigação em Biodiversidade e Recursos Genéticos (CIBIO), InBIO Laboratório Associado Universidade do Porto Vairão Portugal; ^4^ BIOPOLIS Program in Genomics, Biodiversity and Land Planning CIBIO Vairão Portugal; ^5^ Departamento de Biologia, Faculdade de Ciências Universidade do Porto Porto Portugal

**Keywords:** experimental introduction, founder effect, island, *Podarcis siculus*, population genomics, rapid adaptation

## Abstract

Experimental introductions of species have provided some of the most tractable examples of rapid phenotypic changes, which may reflect plasticity, the impact of stochastic processes, or the action of natural selection. Yet to date, very few studies have investigated the neutral and potentially adaptive genetic impacts of experimental introductions. We dissect the role of these processes in shaping the population differentiation of wall lizards in three Croatian islands (Sušac, Pod Kopište, and Pod Mrčaru), including the islet of Pod Mrčaru, where experimentally introduced lizards underwent rapid (~30 generations) phenotypic changes associated with a shift from an insectivorous to a plant‐based diet. Using a genomic approach (~82,000 ddRAD loci), we confirmed a founder effect during introduction and very low neutral genetic differentiation between the introduced population and its source. However, genetic depletion did not prevent rapid population growth, as the introduced lizards exhibited population genetic signals of expansion and are known to have reached a high density. Our genome‐scan analysis identified just a handful of loci showing large allelic shifts between ecologically divergent populations. This low overall signal of selection suggests that the extreme phenotypic differences observed among populations are determined by a small number of large‐effect loci and/or that phenotypic plasticity plays a major role in phenotypic changes. Nonetheless, functional annotation of the outlier loci revealed some candidate genes relevant to diet‐induced adaptation, in agreement with the hypothesis of directional selection. Our study provides important insights on the evolutionary potential of bottlenecked populations in response to new selective pressures on short ecological timescales.

## Graphical abstract

Our study provides unprecedented information on how demographic expansion can help genetically impoverished populations successfully adapt to new environments. Such interplay between demography and selection can have a major role in the evolution of species responding to global changes, such as introduced species and species impacted by climate change.

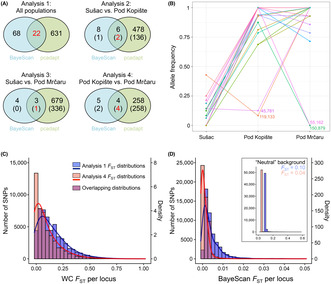

## 1 INTRODUCTION

Studies of “evolution in action” in wild populations have shown that dramatic phenotypic changes can emerge in just a few generations (Hendry, 2016; Messer et al., 2016), for example, in response to environmental perturbations (Campbell‐Staton et al., 2017; Eloy de Amorim et al., 2017; Franks et al., 2016; Grant & Grant, 2002; Winchell et al., 2023) or following species introductions (Colautti & Lau, 2015; Prentis et al., 2008). Species on islands often show particularly fast evolutionary rates compared to mainland populations (Millien, 2006). However, separating evolution due to neutral processes from responses due to selection remains challenging (Keller & Taylor, 2008; Sherpa & Després, 2021). Experimental evolution with controlled field manipulations can help alleviate the challenges via a priori knowledge of the original source population, the time of introduction, and the ability to control the number of founders. Such experimental introductions have provided some of the strongest empirical evidence of rapid phenotypic differentiation across a range of organisms, including anole lizards (Calsbeek & Cox, 2010; Kolbe et al., 2012; Losos et al., 2004; Stuart et al., 2014), peppered moths (Cook et al., 2012; Cook & Saccheri, 2013), and Trinidadian guppies (Reznick & Travis, 2019), but less research has focused on describing the underlying genetic architecture (although see Fraser et al., 2015; Whiting et al., 2022; van der Zee et al., 2022).

Rapid adaptive genetic changes can involve a large number of small‐effect loci or a few loci with strong effects (Stephan, 2016), but the role of many beneficial variants is less likely when selection acts for only short periods of time and when genetic drift is strong (Dlugosch et al., 2015; Messer & Petrov, 2013; Schluter et al., 2021). Under these conditions, experimentally introduced populations are expected to show very limited levels of background differentiation compared to their source population, except at loci showing an extreme level of differentiation (i.e., outliers). However, the random subsampling of the source gene pool can induce rapid differentiation at neutral loci and a low amount of standing genetic variation in the introduced population, which ultimately may reduce its ability to adapt to new selective forces (Dlugosch et al., 2015; Willi et al., 2006). Nevertheless, rapid population growth can also create favorable conditions when mutations accumulate polymorphism much faster than genetic drift can remove them. Identifying genomic signals of selection therefore requires describing the genomic diversity and past demography of the introduced population. The short time‐scale at which these demographic events occur complicates the use of genetic demographic inference methods (Epps & Keyghobadi, 2015; van der Zee et al., 2022), but the distribution of genetic polymorphisms within and among populations contains information about population bottlenecks and expansions.

One documented example of rapid phenotypic changes associated with responses to new ecological conditions is the experimental introduction of Italian wall lizards (*Podarcis siculus*) to tiny islets in the central Mediterranean Sea (Herrel et al., 2008; Nevo et al., 1972), when, in 1971, just five adult pairs were introduced from the islet of Pod Kopište to the nearby Pod Mrčaru (Croatia). In approximately 30 generations, the introduced population reached population densities five times higher than the source population (Herrel et al., 2008). Furthermore, the introduced lizards rapidly developed a suite of traits commonly found in plant‐eating reptiles, including a larger body size, a modified skull shape, wider teeth, and specific gut structures, digestive functions, and microbiota (Lemieux‐Labonté et al., 2022; Taverne et al., 2020, 2021; Wehrle et al., 2020). These phenotypic changes allowed the introduced population to shift from a mainly insectivorous diet (as found in the source population Pod Kopište) to an omnivorous diet, including an important fraction of plant material (Herrel et al., 2008). Some of these phenotypic changes may correspond to a plastic response to different diet contents (Vervust et al., 2010), but if and how they are reflected at the genomic level has never been assessed.

Here, we investigate the role of neutral processes linked to island colonization and adaptive processes linked to the exploitation of a particular niche in shaping the rapid evolution of three *P. siculus* island populations in Croatia: Sušac, Pod Kopište, and Pod Mrčaru. Our study builds on previous research, showing contrasting patterns between the demographic history of populations and their phenotype and ecology. Genetic analyses revealed a common origin among the three populations, but no genetic differentiation between Pod Kopište and Pod Mrčaru (Podnar et al., 2005; Sherpa et al., 2023). On the contrary, lizards inhabiting Sušac and Pod Mrčaru are distinct from the Pod Kopište population at several phenotypic traits, in relation to a herbivorous diet in Sušac and Pod Mrčaru vs. an insectivorous diet in Pod Kopište (Herrel et al., 2008; Taverne et al., 2019, 2020, 2021; Wehrle et al., 2020). We generated a ddRADseq dataset to answer the following questions: (1) Can we detect genomic differentiation between the introduced population in Pod Mrčaru and its source Pod Kopište?; (2) Do genetic diversity and polymorphism frequencies reflect the impact of a founder effect during introduction (related to the extremely small number of founding individuals) and/or a rapid population expansion after introduction (in relation to the high observed population density)?; (3) Can we identify candidate loci underlying the documented shift in diet and phenotypic traits between Pod Mrčaru and Pod Kopište?; (4) Do ecologically similar populations (Sušac and Pod Mrčaru) show analogous patterns of allele frequency change at candidate loci? Answering these questions helps us understand the role of genetic variability and potential directional selection in shaping rapid evolution.

## 2 MATERIALS AND METHODS

### 2.1 Study system and sampling

**FIGURE 1:**
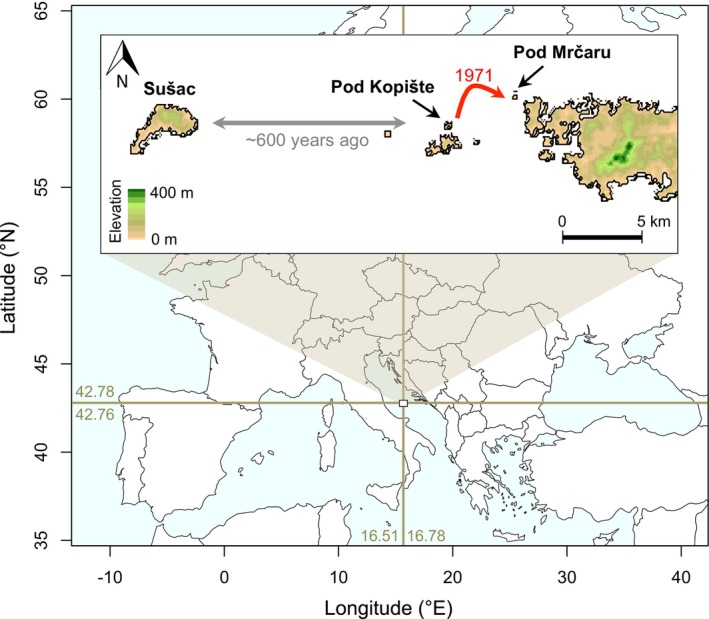
Study area and location of islands. The background map shows the context of the study area within the Adriatic Sea. The insert shows the three islands used in the study (Sušac, Pod Kopište, and Pod Mrčaru). The gray arrow represents the estimated divergence time between Sušac and the islets based on previous genetic analysis (Sherpa et al., 2023). The red arrow represents the known introduction date from Pod Kopište to Pod Mrčaru.

The Italian wall lizard, *Podarcis siculus*, shows a complex biogeographic pattern in the Croatian islands, as their colonization is the result of multiple events of over‐sea dispersal and vicariance driven by Quaternary sea‐level oscillations as well as human‐mediated dispersal (Bonardi et al., 2022; Podnar et al., 2005; Sherpa et al., 2023). The island of Sušac (42.76° N, 16.51° E; surface area: 4.02 km^2^) hosts an endemic clade of wall lizards, which diverged from other conspecific lineages before the penultimate glacial maximum (roughly prior to 200 kya; Podnar et al., 2005; Sherpa et al., 2023). Both genetic and genomic data suggest that lizards in Sušac recently diverged from those in Pod Kopište (16.72° E, 42.76° N; 0.03 km^2^) approximately 600 years ago (Podnar et al., 2005; Sherpa et al., 2023). In 1971, 10 adult lizards from Pod Kopište were experimentally introduced to the tiny islet of Pod Mrčaru (42.78° N, 16.77° E; 0.02 km^2^; Herrel et al., 2008; Nevo et al., 1972). We collected 10 *P. siculus* on each of these three islands in September 2015 (Figure 1). The tail tip (~2 cm) was sampled and stored in 95%–100% ethanol, followed by the immediate release of the specimen. Lizards regenerate tails; therefore, the removal of tail tips carries a very low disturbance (García‐Muñoz et al., 2011).

### 2.2 Genetic data acquisition

DNA extraction was performed using the DNeasy Blood & Tissue kit (Qiagen). Individuals were genotyped using double‐digest restriction‐site‐associated DNA sequencing (ddRADseq). Genomic DNA (300 ng) was digested with 2.4 U of both *Pst*I and *Msp*I (New England Biolabs) in a 30 μL reaction at 37°C for 90 min, followed by a further incubation at 65°C for 20 min. DNA fragments were purified with 1.5 volumes of AMPureXP beads (Agencourt) and subsequently ligated with 180 U of T4 ligase (New England Biolabs) in a 50 μL reaction at 23°C for 60 min and at 20°C for 60 min, followed by a heat‐inactivation step at 65°C (10 min). After purification (1.5 volumes of AMPureXP beads), size selection was performed with BluePippin (Sage Science Inc.), setting the internal fragment size to 250–500 bp. The gel‐eluted fraction was amplified using Phusion High‐Fidelity PCR Master Mix (New England Biolabs) in a 50 μL reaction. PCR conditions followed an initial denaturation at 95°C (3 min), 10 cycles of 95°C (30 s), 60°C (30 s), 72°C (45 s), and a final extension at 72°C (2 min). Amplified products were purified with 1 volume of AMPureXP beads and sequenced on NovaSeq 6000 (150 bp paired‐end, Illumina).

### 2.3 SNP calling and filtering

As there is no available reference genome for *P. siculus*, ddRAD loci were assembled de novo using Stacks v.2.61 (Rochette et al., 2019), following the analytical optimization of de novo parameters as described in Paris et al. (2017). After removing individuals with a low number of individual loci (<100,000), we removed loci with evidence of low coverage (<5 reads) and a high percentage of missing data (>25% in each island). SNPs showing an excess of shared heterozygosity (>0.7) and more than two alleles were removed. We retained SNPs with a minimum minor allele count (MAC = 1) for analysis of the levels of low‐frequency polymorphisms (one random SNP per locus for the number of singletons; all SNPs for neutrality tests), and with MAC = 3 (i.e., at least two individuals) and one random SNP per locus for analysis of population structure and diversity and for analysis of signals of selection.

### 2.4 Population structure and genetic diversity

We calculated genetic diversity indices using the packages hierfstat v.0.04‐22 (Goudet, 2005) and poppr v.2.8.1 (Kamvar et al., 2014) in R v.3.5.2 (R Core Team, 2018). We quantified the following population diversity indices: observed heterozygosity (*H*_o_), expected heterozygosity (*H*_e_), allelic richness (AR), inbreeding coefficient (*F*_IS_), population‐specific *F*_ST_, and the number of private alleles (PA). We determined the 95% confidence intervals (95% CI) of *H*_o_, *H*_e_, AR, *F*_IS,_ and *F*_ST_ by performing 1000 bootstraps over loci. Individual *H*_o_ was also used to test the difference in genetic diversity among populations using ANOVAs and Tukey posthoc statistics (significance: *p* = .05) in R. Diversity indices were estimated using the SNP dataset, excluding the outlier loci identified in the analysis of selection (see below).

Genetic variation among the three island populations was first investigated using a PCA in the R package adegenet v.2.1.1 (Jombart, 2008). We then estimated pairwise Weir and Cockerham's *F*_ST_ (WC *F*_ST_) and 95% CI (1000 bootstraps) using hierfstat v.0.04‐22 (Goudet, 2005) and inferred population structure using ADMIXTURE v1.3 (Alexander et al., 2009). We performed 10 independent runs for *K* = 1 to *K* = 5 possible genetic clusters and obtained the 10‐fold cross‐validation error rates for each *K* using ADMIXTURE (Alexander & Lange, 2011). An ADMIXTURE analysis was performed using all loci, and after removing loci identified as outliers.

### 2.5 Distribution of low‐frequency polymorphisms

To assess the potential effect of our sample sizes, we performed a rarefaction analysis, which showed that good estimates of *H*_e_ and pairwise *F*_ST_ can be obtained from subsample sizes as small as four diploid individuals (Figure S1), supporting the robustness of genetic diversity and differentiation estimates when a large number of SNPs are available (Nazareno et al., 2017). Although desirable, we were unable to perform robust estimations of past and contemporary effective population size, as both the sample size (between 14 and 16 gene copies) and the short time‐scale at which the demographic events occurred (~30 generations) limit the use of both linkage and coalescent approaches (McLaughlin & Winker, 2020; Nunziata & Weisrock, 2018; Robinson et al., 2014; van der Zee et al., 2022). Nevertheless, the frequency distribution of polymorphisms within a population still carries information about recent processes. Specifically, in a growing population, the frequency of singletons increases. Thus, we calculated the total (per population) and average (per individual) number of SNPs with a derived allele count (DAC) of 1 (i.e., singletons) for each of the three island populations. To test the difference in mean number of individual singletons among populations, we run an ANOVA followed by Tukey posthoc tests (significance: *p* = .05) in R.

To detect the impact of a founder effect and a population expansion during the recorded introduction from Pod Kopište to Pod Mrčaru, we calculated Tajima's *D* at each locus. We compared Tajima's *D* for three sets of variants: (1) common polymorphism in all three populations; (2) shared polymorphism in Pod Kopište and Pod Mrčaru (monomorphic in Sušac); and (3) private polymorphism in each population. Tajima's *D* was calculated on the set of loci obtained after removal of loci identified as outliers in the analysis of selection (see below). Under these circumstances, *D* most likely represents the effect of demographic history. We predicted that common polymorphism would reflect the ancestral history in the region, while shared polymorphism in Pod Kopište and Pod Mrčaru and population‐specific polymorphism in Pod Mrčaru would reflect the demographic history of introduced populations. Positive Tajima's *D* values can indicate population contraction, while negative *D* values can indicate recent population expansion after a bottleneck (Simonsen et al., 1995). Tajima's *D* was calculated for loci with ≥5 SNPs using VCFtools v.0.1.16 (Danecek et al., 2011), and the 95% CI of the mean was calculated from 1000 bootstraps over loci.

### 2.6 Identification of highly differentiated SNPs

The identification of candidate loci was performed using two methods: the *F*_ST_‐based method employed in BayeScan v.2.1 (Foll & Gaggiotti, 2008) and the Principal Component Analysis (PCA) method employed in the R package pcadapt v.4.3.3 (Luu et al., 2017). For both approaches, we performed four analyses, contrasting allele frequencies (BayeScan) or individual genotypes (pcadapt) among the three island populations and between pairs of populations. The four analyses were: (1) All populations; (2) Sušac vs. Pod Kopište; (3) Sušac vs. Pod Mrčaru; and (4) Pod Kopište vs. Pod Mrčaru. For BayeScan analyses, the Markov Chain Monte Carlo (MCMC) algorithm was run for 1000,000 iterations following a burn‐in period of 100,000, with the proposal distributions for parameters adjusted by 20 short pilot runs of 5000 iterations. The final sample size was 100,000 iterations (one MCMC sampled every 10 iterations). For pcadapt, we imputed missing genotypes. To avoid inflating the differentiation signal between populations, missing genotypes were imputed by the median locus genotype among individuals using the R package tidyverse v.1.3.2 (Wickham et al., 2019). Highly differentiated SNPs were detected using the two first PCs, and *p*‐values were estimated using the Mahalanobis distance. Multiple comparisons were accounted for by estimating the false‐discovery rate (FDR) using *Q*‐values < 0.01, generating a list of highly differentiated SNPs for each method and analysis. For pcadapt, *Q*‐values were computed using the R package qvalue v.2.6.0 (Storey et al., 2015).

### 2.7 Differentiation at neutral and outlier loci

Highly differentiated SNPs identified by both BayeScan and pcadapt analyses were considered to occur on significant outlier loci; thus, we included loci that overlapped between the two methods but not necessarily among the four analyses (1, all three populations; 2–4, pairwise comparisons). Genetic differentiation at outlier loci was quantified using the allele frequency difference (AFD) between populations by assessing the change in allele frequency among populations. We assessed whether the Pod Mrčaru population shows limited differentiation from its source, except at very few loci showing an extreme level of differentiation. To do this, we compared the distributions of per locus *F*_ST_ estimated between all three populations with the distributions of per locus *F*_ST_ estimated between only the Pod Kopište and the Pod Mrčaru populations. The BayeScan method assumes a model analogous to the empirical history of the studied island populations, where the current subpopulations have a shared history from a common migrant gene pool (a nonstructured ancestral population) (see Podnar et al., 2005; Sherpa et al., 2023), and where the difference in allele frequency between the ancestral pool and each subpopulation (population demography) is measured as a population‐specific component shared by all loci (Foll & Gaggiotti, 2008). However, neutral loci can vary widely in their amount of genetic differentiation, especially in the case of non‐panmictic populations. We therefore also computed the neutral *F*_ST_ distributions using locus‐specific WC *F*_ST_.

### 2.8 Prediction of the functional effect of identified outlier loci

The consensus sequence of significant outlier loci was exported from the Stacks catalog, which was used to perform a preliminary assessment of the potential adaptive function of the identified loci. Because the reference genome for *P. siculus* is not available, contigs of these catalog loci were aligned to the closely related *Podarcis muralis* (Salvi et al., 2021; Yang et al., 2021; PodMur_1.0; GenBank assembly accession: GCA_004329235.1; Andrade et al., 2019) using minimap2 (Li, 2018). Subsequent alignments were converted to BAM format using Samtools v.1.16.1 (Li et al., 2009). We discarded poor alignments, including loci that aligned to multiple locations in the genome and those with poor alignment scores (<40). We then used stacks‐integrate‐alignments (Paris et al., 2017) to add positional information, followed by using the Stacks populations program to write a VCF with the SNP information for the selected outlier loci. To classify the gene‐level effects of potentially adaptive loci, we used the Variant Effect Predictor Tool in Ensembl (VEP; McLaren et al., 2016). For loci marked as intergenic by VEP, we explored if there were any protein‐coding genes further downstream or upstream by intersecting a BED file containing 10 kbp windows flanking both sides of the alignment position with the GFF annotation file.

## 3 RESULTS

### 3.1 ddRADseq data assembly

**FIGURE 2:**
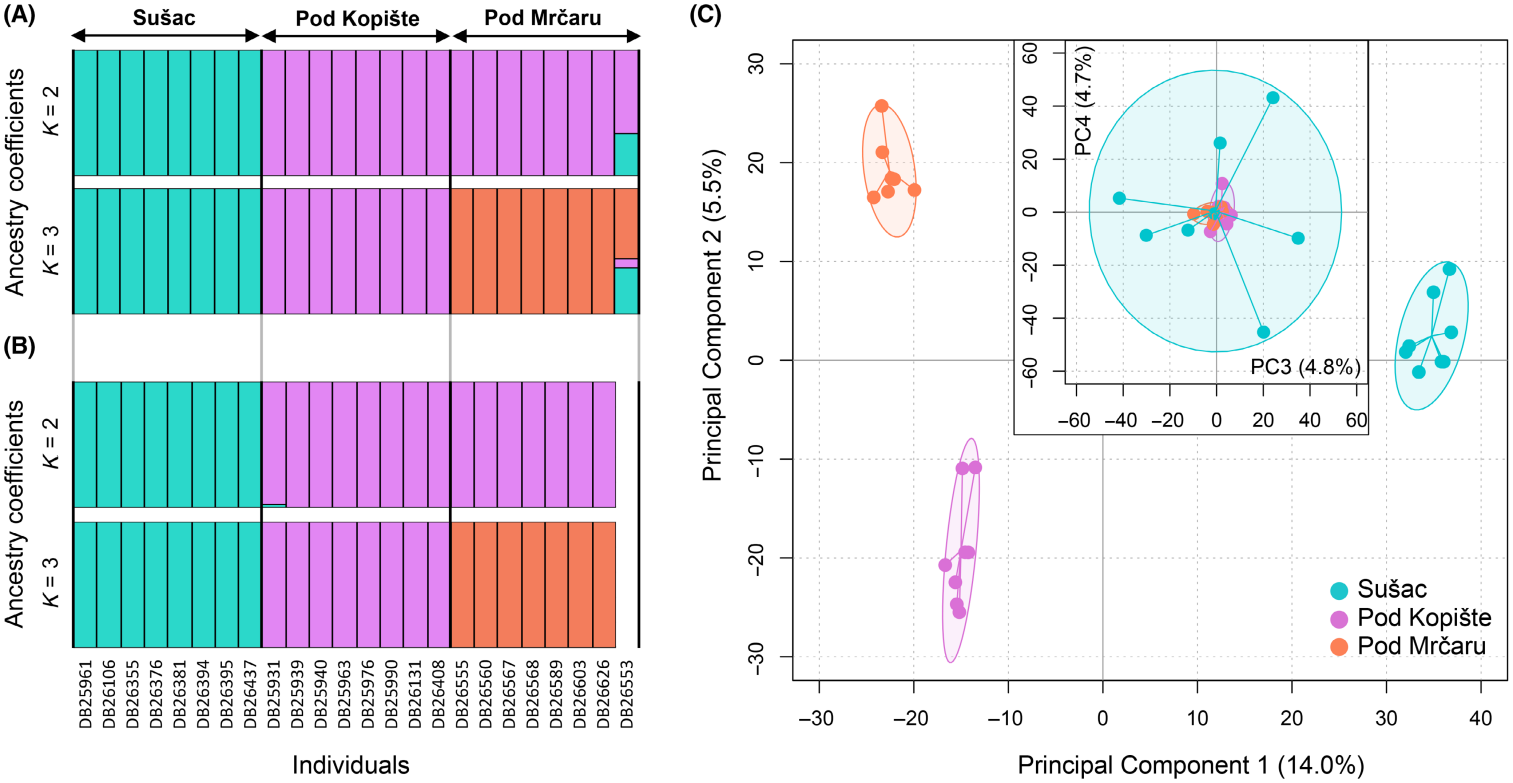
Genetic differentiation among islands. Individual ancestry estimated from ADMIXTURE analysis for *K* = 2 and *K* = 3, including: (A) the 24 individuals genotyped at 54,050 SNPs, including the significant outlier loci; (B) the 23 individuals genotyped at 52,470 SNPs after excluding the 29 outlier loci and the single admixed individual as shown in A; (C) Principal Component Analysis (PCA) summarizing genetic variation among individuals (same SNP dataset as used in B) on the four first PCs of the PCA (29% of total variance).

Approximately 225 million reads were obtained for a total of 30 individuals, with 7.5 ± 4.2 (SD) million high‐quality reads per individual on average (Table S1). We assembled an average of 170 ± 19 thousand loci per individual after removing two individuals within each population, which evidenced fewer than 100,000 loci. The variant calling and SNP filtering for the remaining 24 individuals resulted in 83,487 loci (340,477 SNPs) present in 75% of individuals in each population. Genotypes called from low sequencing coverage (<5) were removed (626 loci), resulting in 82,861 loci (331,827 SNPs). SNPs showing an excess of shared heterozygosity (2154 SNPs distributed over 457 loci) were further filtered, resulting in 329,673 SNPs distributed over 82,404 loci. After removing loci with low minor allele counts (MAC < 3) and selecting one random SNP per locus, we retained 54,050 loci with a read coverage of 25.4 ± 7.1 (mean ± standard deviation, SD) and 6.4 ± 5.2% of missing data per sample (Table S1). A preliminary ADMIXTURE analysis using this dataset revealed one highly admixed individual in Pod Mrčaru, potentially indicating very recent gene flow with Sušac (Figure 2A). This individual was therefore removed for all subsequent analyses. After re‐filtering loci based on missing data percentage, we obtained a dataset of 73,876 loci (from 82,404 loci; MAC = 1) that was used for analyses of frequency polymorphisms, and a dataset of 52,499 loci (from 54,050 loci; MAC = 3) that was used for population structure and diversity analyses and for the identification of genomic signals of selection.

### 3.2 Genetic variability and divergence of island populations

The overall rate of among‐population genetic differentiation was low, as determined by an optimal number of *K* = 1 (lowest cross‐validation error rate; Figure S2). Nonetheless, further inspection of individual‐level co‐ancestry based on higher values of *K* revealed that Sušac individuals clustered in a separate genetic group from individuals from Pod Kopište and Pod Mrčaru. Pod Kopište and Pod Mrčaru were further clustered into two different groups (Figure 2B). Accordingly, WC pairwise *F*_ST_ were higher between Sušac and Pod Mrčaru (*F*_ST_ = 0.134, 95% CI = [0.132–0.136]) and Sušac and Pod Kopište (*F*_ST_ = 0.106, 95% CI = [0.104–0.108]) than between Pod Kopište and Pod Mrčaru (*F*_ST_ = 0.036, 95% CI = [0.034–0.037]). The PCA differentiated the three islands on the two first PCs (Figure 2C). PC1 (14.0% of variance) accounted for the differentiation between Sušac and the two other populations; PC2 (5.5% of variance) separated Pod Kopište and Pod Mrčaru; and PC3 and PC4 (together accounting for 9.5% of variance) revealed high genetic variability among the Sušac individuals.

**FIGURE 3:**
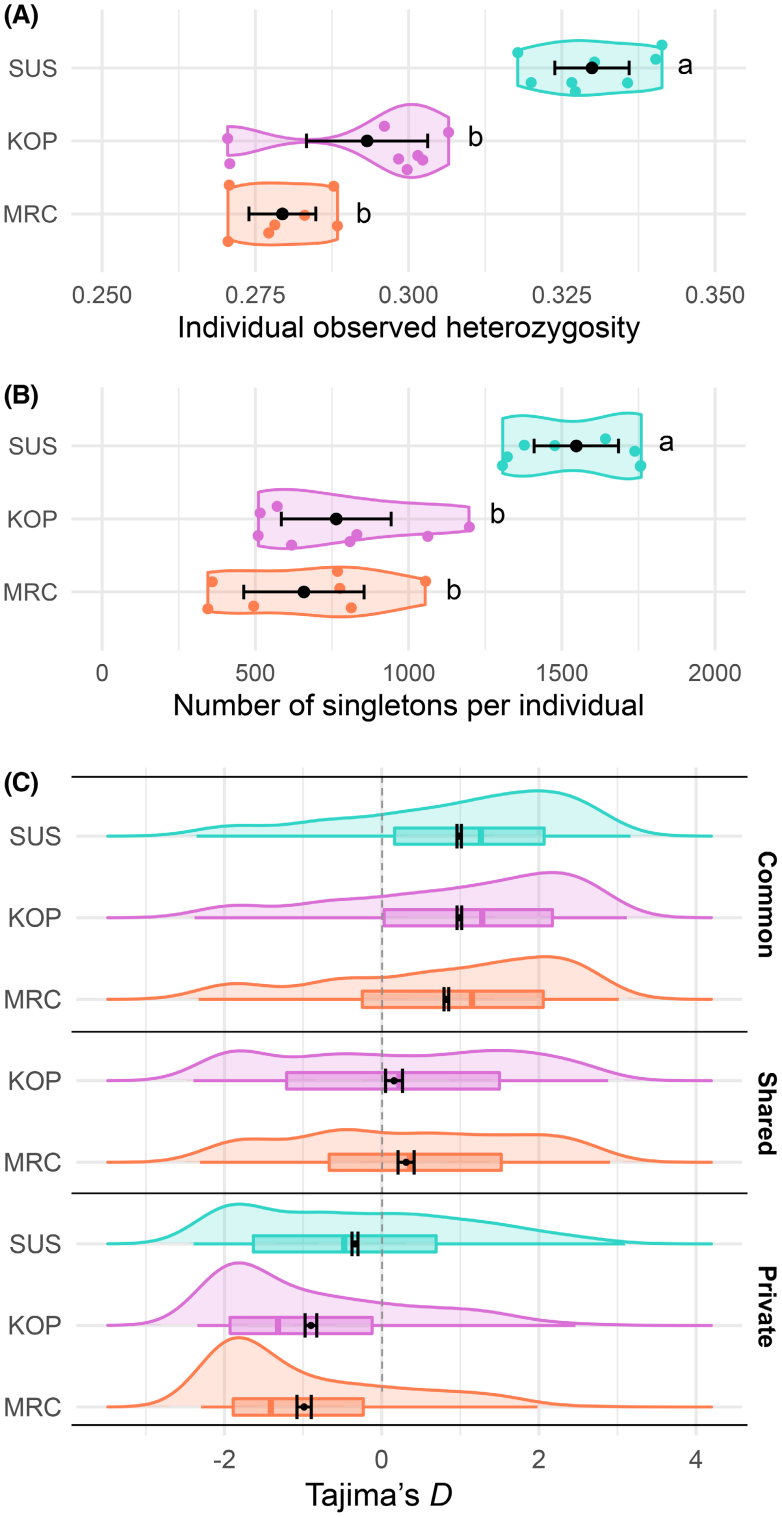
Distribution of individual genetic diversity and low‐frequency polymorphisms among populations. (A) Observed individual heterozygosity among populations (MAC = 3; 52,470 SNPs). (B) Number of uniquely represented derived alleles per individual among the 23,097 singleton variants (MAC = 1; 73,847 SNPs). (C) Tajima's *D* values among loci for polymorphic loci in all three islands (common), in only Pod Kopište and Pod Mrčaru (shared), and in only one island (private). Error bars: 95% confidence intervals. Letters (a, b) in A and B: results of Tukey posthoc statistics testing pairwise differences of means between populations (significance: *p* = .05).

The within‐population genetic diversity varied significantly among populations, with population heterozygosity (observed heterozygosity, *H*_o_; expected heterozygosity, *H*_e_), allele richness (AR), and the number of private alleles (PA) showing a gradual decrease from Sušac to Pod Kopište to Pod Mrčaru (Table 1). Inbreeding coefficients (*F*_IS_) were all significantly different from zero but only slightly positive. Population‐specific *F*_ST_ was not different from zero in Sušac, suggesting that the genetic variability of this population does not differ from the ancestral population, while population‐specific *F*_ST_ was significantly positive in the two islets, indicative of genetic drift, especially in Pod Mrčaru (Table 1). The average *H*_o_ per individual was significantly different between populations (ANOVA, *F*_2,20_ = 45.59, *p* < .001), being highest in Sušac (0.330 ± 0.003 (SE)), while *H*_o_ was not significantly different between Pod Kopište (0.293 ± 0.005) and Pod Mrčaru (0.279 ± 0.003) (Tukey posthoc test; Figure 3A).

**TABLE 1:** Quantification of genetic diversity among islands. Sample sizes are represented in column *N*.

Island	*N*	*H*_o_	*H*_e_	*F*_IS_	*F*_ST_	AR	PA	NS
Sušac	8	0.329 [0.327–0.331]	0.364 [0.362–0.365]	0.075 [0.072–0.078]	−0.002 [−0.006–0.002]	1.878 [1.876–1.880]	5446	12,377
Pod Kopište	8	0.294 [0.292–0.296]	0.317 [0.316–0.319]	0.052 [0.049–0.055]	0.125 [0.122–0.128]	1.790 [1.787–1.793]	371	6111
Pod Mrčaru	7	0.279 [0.277–0.281]	0.297 [0.295–0.300]	0.039 [0.035–0.042]	0.181 [0.178–0.184]	1.739 [1.735–1.742]	245	4609

*Note*: Diversity indices: observed heterozygosity (*H*_o_); expected heterozygosity (*H*_e_); inbreeding coefficient (*F*_IS_); population‐specific differentiation (*F*_ST_); allelic richness (AR); private alleles (PA); number of singletons (NS). Confidence intervals (CI): 95% CI based on 1000 bootstraps over loci. Genetic diversity measures were estimated using the 52,470 SNP dataset. The number of singletons (NS) was calculated using the 73,847 SNP dataset, of which a total of 23,097 SNPs were singletons.

### 3.3 Analysis of rare and low‐frequency polymorphisms

Among the quantified singletons (DAC = 1) among the three populations, 53% were carried by the Sušac population, whereas only 27% and 19% were carried by the Pod Kopište and Pod Mrčaru populations, respectively (Table 1). Given the total number of polymorphic sites in Sušac (58,588), in Pod Kopište (46,200), and in Pod Mrčaru (41,007) among 73,847 SNPs, the proportion of singletons was highest in Sušac (21%) but very similar between Pod Kopište (13%) and Pod Mrčaru (11%). The average number of singletons per individual was significantly different among populations (ANOVA, *F*_2,20_ = 31.39, *p* < .001), which was highest in Sušac (1547 ± 70 (SE)), but was not significantly different between Pod Kopište (764 ± 91) and Pod Mrčaru (658 ± 100) (Figure 3B).

Population‐level summary statistics differed between common polymorphisms in all three populations, shared polymorphisms between Pod Kopište and Pod Mrčaru (monomorphic in Sušac), and private polymorphisms (Figure 3C). For the common polymorphisms, the average Tajima's *D* was positive in Sušac (*D* = +0.99, 95% CI = [0.96–1.01]), Pod Kopište (*D* = +0.99, 95% CI = [0.96–1.02]), and Pod Mrčaru (*D* = +0.82, 95% CI = [0.79–0.85]), consistent with a population contraction experienced by the lizards in the region. For the shared polymorphisms, the mean Tajimia's *D* was positive but lower in Pod Kopište (Figure 3C; *D* = +0.16, 95% CI = [0.05–0.27]) compared to Pod Mrčaru (*D* = +0.31; 95% CI = [0.21–0.41]), suggesting an impact of genetic drift in the latter population. Tajima's *D* for the private polymorphisms in Pod Kopište (*D* = −0.90, 95% CI = [−0.97–0.83]) and in Pod Mrčaru (*D* = −1.07, 95% CI = [−1.07–0.89]) were all negative, consistent with a population expansion after a recent bottleneck. As a comparison, Tajima's *D* for private polymorphisms in Sušac was closer to equilibrium (*D* = −0.34, 95% CI = [−0.38–0.30]).

### 3.4 Outlier analysis to detect candidate loci for selection

**FIGURE 4:**
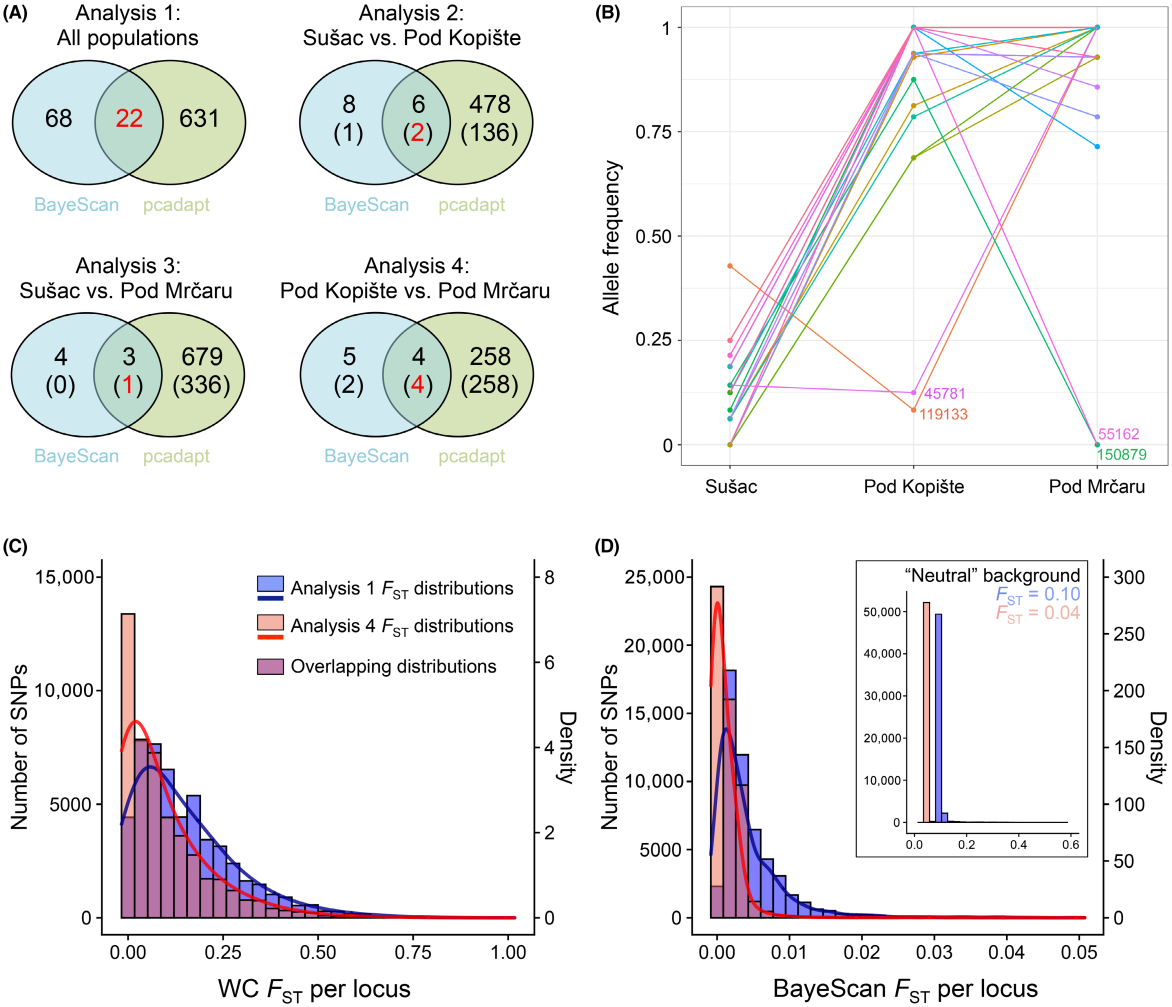
Genomic signals of selection among populations. (A) Venn diagram showing the overlap between the highly differentiated SNPs identified by BayeScan (blue circle) and pcadapt (green circle) for the four analyses. For comparisons 2–4, the upper number indicates the total number of SNPs, and the bottom number indicates the unique number of SNPs not detected by analysis 1 (i.e., significant outlier SNPs, in red). (B) Schematic representing the allele frequencies of the 29 outlier SNPs among the three island populations (frequency of the allele that is the rarest in Sušac). The ID of loci detected by the analysis of Pod Kopište vs. Pod Mrčaru is indicated. Distributions of (C) locus‐specific WC *F*_ST_ and (D) average BayeScan locus‐population‐specific *F*_ST_ for analyses 1 and 4. In (D), the graph is centered on low values normalized to zero by subtracting the modeled neutral background (see insert).

The analysis including all three populations revealed 68 and 631 highly differentiated SNPs (*Q*‐values ≤ 0.01) for BayeScan and pcadapt, respectively, of which 22 were detected by both methods and were therefore considered significant outliers (Figure 4A). Pairwise comparisons (Sušac vs. Pod Kopište; Sušac vs. Pod Mrčaru; Pod Kopište vs. Pod Mrčaru) detected a further set of 12 overlapping outliers, of which five were also detected by the three‐population analysis, but only four loci were detected between the introduced population in Pod Mrčaru and its source in Pod Kopište. Overall, we generated a list of 29 significant outlier loci (Figure 4B). A high‐quality alignment (≥40) to the genome of *P. muralis* was obtained for 15 of the 29 significant outlier loci (Table 2). Seven loci were classified as intergenic (>10 kbp away from genes), and one locus was a downstream variant (~1200 bp) but was in a gene that is not functionally annotated (ENSPMRG00000019065). The seven remaining loci were all functionally annotated and included six loci in intronic regions, and one locus was an upstream variant (~800 bp).

**TABLE 2:** List of significant outlier loci with high‐quality alignment.

Locus ID	Analysis				Alignment				Prediction and annotation		
	Among islands	Sušac vs. PK	Sušac vs. PM	PK vs. PM	Chromosome	Position	*q*	Number of SNPs	VEP result	*P. muralis* gene ID	Gene description
6910	×	×	×		NC_041330.1 (chrZ)	32627886	56	5	Downstream (1208 bp)	ENSPMRG00000019065	Novel gene
26500		×			NC_041325.1 (chr14)	27802439	59	3	Intergenic		
26543	×				NC_041317.1 (chr6)	97058925	60	9	Intergenic		
45781				×	NC_041330.1 (chrZ)	36303140	60	3	Intergenic		
55162				×	NC_041330.1 (chrZ)	36296785	60	3	Intergenic		
60840	×				NC_041317.1 (chr6)	24386790	60	1	Intronic	ENSPMRG00000007382	RPAP2
75301	×	×			NC_041312.1 (chr1)	33774232	60	13	Intronic	ENSPMRG00000008942	SLC25A21
119133				×	NC_041328.1 (chr17)	30724716	40	22	Upstream (777 bp)	ENSPMRT00000035880	POSTN
120515	×		×		NC_041314.1 (chr3)	68304198	60	5	Intergenic (10,216 bp)	ENSPMRG00000010821	TIAM2
122458	×				NC_041313.1 (chr2)	104687192	60	7	Intronic	ENSPMRG00000013607	FAM3D
133726	×	×			NC_041320.1 (chr9)	37118335	60	2	Intergenic		
142246	×				NC_041323.1 (chr12)	21832221	40	5	Intronic	ENSPMRT00000021323	ABI1
153860	×				NC_041317.1 (chr6)	80888946	60	1	Intergenic		
157055	×				NC_041320.1 (chr9)	33027141	60	1	Intronic	ENSPMRT00000017049	PALLD
157640	×				NC_041313.1 (chr2)	40816478	60	6	Intronic	ENSPMRG00000016255	DNAH9

*Note*: Alignment to the *P. muralis* genome: Chromosome ID (chromosome number); position of alignment; Quality of alignment (*q*); number of SNPs per locus. Annotation: Variant Effect Predictor (VEP) result (protein‐coding gene or <10 kbp distant for intergenic regions); gene description.

Abbreviations: PK, Pod Kopište; PM, Pod Mrčaru.

The absolute difference in allele frequency (AFD) at significant outlier loci revealed high differentiation among the three populations, ranging between 0.75 and 1.00 (Figure 4B). Only four of the loci showed fixed differences in at least two populations due to high polymorphism in Sušac. The number of fixed alleles increased from Sušac (5) to Pod Kopište (15) to Pod Mrčaru (22). The average differentiation was higher between Sušac and Pod Kopište (AFD = 0.79 ± 0.19), and between Sušac and Pod Mrčaru (AFD = 0.80 ± 0.20), than between Pod Kopište and Pod Mrčaru (AFD = 0.19 ± 0.31). The average locus‐specific WC *F*_ST_ (*F*_ST_ = 0.801, 95% CI = [0.773–0.829]) and locus‐specific BayeScan *F*_ST_ (*F*_ST_ = 0.362, 95% CI = [0.343–0.380]) were high for the 29 identified outlier loci, as compared to the average differentiation at 52,470 neutral loci (WC *F*_ST_ = 0.155, 95% CI = [0.154–0.156]; BayeScan *F*_ST_ = 0.097, 95% CI = [0.096–0.097]). The distributions of locus‐specific *F*_ST_ revealed a higher proportion of SNPs with low differentiation and a lower proportion of SNPs with intermediate differentiation in the Pod Kopište vs. Pod Mrčaru analysis compared to the among‐populations analysis (Figure 4C,D).

## 4 DISCUSSION

The geographic isolation and the strong selective forces acting on insular biotas have promoted spectacular radiation and rapid evolutionary adaptations (Jønsson et al., 2012; Lapiedra et al., 2021; Losos & Ricklefs, 2009). Still, small islands are expected to harbor reduced population sizes and genetically impoverished populations due to colonization history, reduced resources, increased genetic drift, and restricted gene flow (Frankham, 1998). The observed differences in population genetic diversity between Sušac and the two islets support the relationship between a small effective population size and a small island area. Indeed, Sušac is larger (4.02 km^2^) than Pod Kopište (0.03 km^2^), and Pod Mrčaru, which is even smaller (0.02 km^2^). Furthermore, the two islet populations appeared to show a loss of rare alleles and a reduced gene pool compared to Sušac lizards. In principle, such small populations should have reduced standing genetic variation upon which selective forces can act (Dlugosch et al., 2015; Willi et al., 2006). Our study reveals that even with extreme founder effects (only five adult pairs introduced in Pod Mrčaru, originating from an already genetically impoverished population), potentially adaptive alleles can emerge over the course of just a few generations in introduced populations.

### 4.1 Genetic signatures of population history

The genetic differentiation among populations reflects population history in terms of the known colonization history. The genetic structure of the three populations confirms the ancestral relationship between Pod Kopište and Pod Mrčaru (Herrel et al., 2008; Nevo et al., 1972), and the level of population differentiation between Sušac and the two islets compliments the hypothesis that they diverged from Sušac several hundred years ago (Sherpa et al., 2023). Similar to the cumulative effects of founding events during spatial expansions (Excoffier et al., 2009), processes of linear colonization events can lead to a gradual turnover in allele frequency from the initial source island to the stepping‐stone island and then to the most recently colonized island (Excoffier et al., 2009; Le Corre & Kremer, 1998). The proportion of fixed alleles in Sušac (7%), Pod Kopište (15%), and Pod Mrčaru (22%) is in agreement with recurrent founding bottleneck effects.

The within‐population diversity and the distribution of low‐frequency polymorphisms also reflect the population history of Pod Mrčaru lizards in terms of population numbers. A low founding population size due to a strong bottleneck event was anticipated, given the small number of introduced individuals. In addition, the population diversity estimates revealed a slight reduction of heterozygosity and allelic richness and a higher population‐specific *F*_ST_ in Pod Mrčaru compared to its source in Pod Kopište. This reduction in standing genetic variation could have negatively impacted the establishment of wall lizards in Pod Mrčaru (Dlugosch et al., 2015). However, despite clear evidence of a founder effect, the introduced population showed a similar number of private alleles and individual number of singletons compared to its source, which might be caused by the rapid accumulation of new variants in a very short period of time (~30 generations). This is supported by Tajima's *D* statistics, which are consistent with a scenario of population expansion following a recent bottleneck. Our genetic results are in line with empirical observations documenting that the introduced lizards reached a higher density compared to the source population (Herrel et al., 2008). Severe founder effects do not necessarily prevent populations from establishing and subsequently undergoing sudden demographic expansion, as is often observed in invasive species (Dlugosch & Parker, 2008; Ficetola et al., 2008).

### 4.2 Potential candidate genes to diet‐induced adaptation

The analysis aimed at identifying signals of selection revealed 29 outlier loci. To extend these analyses further and to reduce the false positive rate, we performed a functional annotation of these loci using alignments to the genome of the congeneric *P. muralis* (the reference genome is not available for *P. siculus*). Despite generally high conserved synteny in *Podarcis* lizards (Gabrielli et al., 2023), 14 of the outlier loci did not align with the *P. muralis* genome. However, by their nature, adaptive loci may not necessarily be syntenic (Larkin et al., 2009; Yeaman, 2013). Out of the 15 high‐quality alignments, we discuss the potential adaptive roles of the seven functionally annotated loci, as those detected within large intergenic regions might correspond to spurious selection signals (i.e., the impact of the genetic bottleneck and subsequent drift).

One locus (locus 119133) showed particularly striking shifts in allele frequency: 0.43 in Sušac, 0.08 in Pod Mrčaru, and 1 (i.e., completely fixed) in Pod Kopište. This locus is positioned approximately 800 bp upstream and is therefore likely in linkage with the gene periostin (POSTN). This protein has various roles in a range of tissues, with a major role in bone development (Conway et al., 2014). Periostin is highly expressed in the periosteum, the layer of connective tissue surrounding bone, and is responsible for bone growth required for an increase in bone diameter and thus bone strength. Periostin is also highly expressed in the periodontal ligament surrounding teeth and is responsible for attaching them to the underlying bone (Conway et al., 2014). This gene annotation may therefore be relevant to diet‐based adaptation. In fact, previous research has evidenced that lizards from Pod Mrčaru and Pod Kopište have significant differences in head morphology and bite force that underlie, in part, differences in diet (Herrel et al., 2008; Taverne et al., 2019, 2020, 2021). This locus therefore represents an interesting potential candidate for diet‐induced adaptation.

The remaining six loci were all found to be intronic. The identification of significant outlier variants located within genes means that they still might be linked to or have unknown regulatory importance to gene function; thus, we discuss those with potential significance. The detected genes include a solute carrier (SLC25A21), a homolog of the OCD1 protein involved in intracellular organic acid catabolism and metabolism (Fiermonte et al., 2001). Diet‐induced variation in the expression of this gene has been found in the fruit fly (Ng'oma et al., 2020), as well as in response to plant protein‐based diets in zebrafish and Atlantic salmon (Dhanasiri et al., 2020). We also identified ABI1, an actin cytoskeleton adaptor molecule that plays a role in macropinocytosis (nutrient uptake and degradation) (Dubielecka et al., 2010); and FAM3D, which regulates glucose and lipid metabolism and is affected by nutritional status (de Wit et al., 2012).

Overall, most of the strong allele frequency differences at outlier loci were observed between Sušac and the two islets, while only subtle frequency changes were observed between Pod Mrčaru and Pod Kopište. However, the lizard's diet is mostly insectivorous in Pod Kopište, while it is more omnivorous in both Sušac and Pod Mrčaru (Herrel et al., 2008; Taverne et al., 2019). The discrepancy between genomic responses and dietary observations might occur for several reasons. First, some of these loci may be unrelated to diet. We highlight that Pod Mrčaru and Pod Kopište are tiny islets that probably experience other strongly similar selective pressures (e.g., microclimate, productivity, sexual selection; Donihue et al., 2016, 2020; Muraro et al., 2022; Sacchi et al., 2015). Second, the large phenotypic differences between the islets could be related to phenotypic plasticity, as might be expected in this generalist species, which occupies a wide niche (Vervust et al., 2010; Zuffi & Giannelli, 2013). Third, Sušac and Pod Mrčaru might show distinct genomic responses to similar selective pressures. Finally, identifying outlier loci remains a major methodological challenge in evolutionary analyses. To reduce error rates, we combined the results of two selection detection methods (de Villemereuil et al., 2014; Leigh et al., 2021). Nonetheless, we cannot fully discard the possibility of false positives, as allele frequency changes due to genetic drift can be hard to distinguish from those driven by selection in bottlenecked populations (Foll & Gaggiotti, 2008; Poh et al., 2014; Shultz et al., 2016).

### 4.3 A low genomic signal of selection

There is increasing evidence for rapid genetic adaptation in colonizing populations (e.g., Laurentino et al., 2020; Marques et al., 2018; Schluter et al., 2021), and our detection of selection signals could support the idea that fast evolutionary changes can also occur after strong bottlenecks, especially when followed by rapid population recovery. The analysis of signals of selection only revealed a clear signature of potential diversifying selection in a small fraction of the 52,499 analyzed loci (0.06%). Using ddRAD to identify loci under selection has been criticized, as the number of loci genotyped generally covers a small part of the genome, potentially missing the targets of selection (Hoban et al., 2016). For instance, Lowry et al. (2017) estimated that 20,000 RAD loci only have sufficient power to cover approximately 7% of the total genome for a species with a 3 Gb genome span and moderate linkage. The extent of linkage has not been characterized in *Podarcis* lizards. However, the genome size of *Podarcis* lizards is 1.5–1.6 Gb (Andrade et al., 2019; Gabrielli et al., 2023), and we obtained ~82,000 RAD loci. The high number of loci, considering genome size, clearly increases the probability of obtaining useful genome‐wide information. This is confirmed by the good coverage of the RAD loci on the genome of *P. muralis* (Figure S3). Nonetheless, we are aware that the few candidate loci we report likely do not represent the full extent of genes under selection given the sparseness of our loci across the genome.

A handful of loci showed evidence of strong allelic shifts between ecologically divergent populations and could therefore be indicative of rapid genetic adaptation. If the extreme changes in phenotypic traits observed between Pod Mrčaru and Pod Kopište involve selection, then the presence of potentially adaptive alleles could derive from new mutations, as suggested by differentially fixed alleles in Pod Mrčaru and Pod Kopište, even though our sample sizes may have limited our capacity to detect low‐frequency pre‐existing alleles in Pod Kopište (i.e., rare alleles). Nonetheless, the role of gene flow is an alternative hypothesis to be further considered given the genetic admixture and diet similarities between the lizards in Pod Mrčaru and Sušac. The overall low signal of selection could reveal that a small number of loci have large effects on the observed phenotypic differences. Nevertheless, the statistical methods used here perform better at detecting highly differentiated loci compared to small‐effect loci. Subtle allele frequency change at small‐effect loci may be further confounded by the effects of founding events and genetic drift (Haasl & Payseur, 2016; Messer & Petrov, 2013), which are likely very strong in this system. These stochastic events can also induce rapid phenotypic divergence between introduced and source populations (Keller & Taylor, 2008; Kolbe et al., 2012; Sendell‐Price et al., 2020). Yet, the observed *F*_ST_ distribution supports that the overall neutral background of differentiation between Pod Mrčaru and Pod Kopište is very low. In fact, the identification of outlier loci at genes that could play a role in diet‐induced adaptation is in agreement with the idea that a combination of phenotypic plasticity and directional selection underlies the ecological and phenotypic divergence of the Pod Mrčaru population (Herrel et al., 2008; Vervust et al., 2010). A reciprocal transplant experiment would be a great approach to elucidating the role of these processes on the observed phenotypic differences between lizards on these islands (Donihue et al., 2023).

## 5 CONCLUSION

Experimental introductions provide a playground to test ecological and evolutionary hypotheses, but few studies have quantified the role of population history and the potential mode and action of natural selection. Our genomic analyses identified a small number of candidate loci that may contribute to the rapid evolution of *Podarcis siculus* in this system. Despite a strong genetic bottleneck, rapid population growth could have played a key role in providing the genetic substrate for evolution to act upon, enabling adaptation in genetically impoverished populations. This interplay between demography and selection can have a major role in the evolution of species responding to global changes, such as invasive species and species impacted by climate change.

## AUTHOR CONTRIBUTIONS

**Stéphanie Sherpa:** Formal analysis (equal); investigation (lead); writing – original draft (equal); writing – review and editing (equal). **Josephine R. Paris:** Formal analysis (equal); writing – original draft (equal); writing – review and editing (equal). **Iolanda Silva‐Rocha:** Conceptualization (supporting); investigation (equal); writing – review and editing (equal). **Viola Di Canio:** Investigation (supporting); writing – review and editing (supporting). **Miguel Angel Carretero:** Conceptualization (equal); writing – review and editing (equal). **Gentile Francesco Ficetola:** Conceptualization (equal); writing – review and editing (equal). **Daniele Salvi:** Conceptualization (equal); writing – review and editing (equal).

## CONFLICT OF INTEREST STATEMENT

The authors declare that they have no conflict of interest to disclose.

## Supporting information


Appendix S1

## Data Availability

Individual ddRAD sequences are available under study accession PRJEB65979 in the European Nucleotide Archive repository (http://www.ebi.ac.uk/ena) with sample accession numbers provided in Table S1. The datasets supporting the results of this article are available in the Dryad digital repository (https://doi.org/10.5061/dryad.rxwdbrvg2).
