# Supplementary material for: Genetic depletion does not prevent rapid evolution in island‐introduced lizards
Source: Ecol Evol. 2023 Nov 27;13(11):e10721. doi: 10.1002/ece3.10721 (PMC10682264; doi:10.1002/ece3.10721)
Supplement: Supplementary file 1 — Appendix S1 [file ECE3-13-e10721-s001.pdf]

Supporting Information

Supplementary Tables

Table S1 ddRADseq data statistics for the 30 individuals. Results of the Stacks pipeline: Total reads: number of demultiplixed reads; Merged stacks: number of individuals stacks; Individual loci: number of paired-end loci matching a catalog locus; Dataset: percentage of missing data in the 54,050 SNP dataset (*populations* results). Cov: mean coverage among loci. Six individuals with less than 100,000 stacks were removed (in red) for analyses. Accession: sample accession number in the European Nucleotide Archive repository (study accession PRJEB65979).

Sample ID	Population	Accession	Total reads	Merged stacks		Individual loci		Dataset	
				Number	Cov	Number	Cov	Missing	Cov
DB25931	Pod Kopište	ERS16339480	13381724	185,603	29.71	178,073	33.58	0.02	37.46
DB25939	Pod Kopište	ERS16339481	10142707	181,086	23.04	174,658	25.79	0.02	28.39
DB25940	Pod Kopište	ERS16339482	8297612	169,846	19.60	163,996	21.41	0.03	23.22
DB25944	Pod Kopište		193988	1,014	10.44				
DB25963	Pod Kopište	ERS16339484	11101397	189,365	24.12	182,129	27.65	0.02	30.86
DB25976	Pod Kopište	ERS16339485	7211191	154,321	18.30	149,469	19.64	0.07	20.59
DB25990	Pod Kopište	ERS16339486	5176964	137,403	14.10	132,778	14.96	0.16	15.58
DB26126	Pod Kopište		2666298	78,541	10.23				
DB26131	Pod Kopište	ERS16339488	11247082	174,107	26.71	168,067	29.49	0.04	32.08
DB26408	Pod Kopište	ERS16339494	4664815	129,866	12.61	125,680	13.25	0.20	13.47
DB26553	Pod Mrčaru	ERS16339496	10312757	194,290	20.61	185,082	24.02	0.16	26.07
DB26555	Pod Mrčaru	ERS16339497	10159794	182,309	22.91	175,494	25.54	0.02	27.97
DB26560	Pod Mrčaru	ERS16339498	12826321	187,570	28.90	180,793	32.88	0.02	36.43
DB26567	Pod Mrčaru	ERS16339499	5738261	147,799	14.16	142,377	15.08	0.12	15.71
DB26568	Pod Mrčaru	ERS16339500	8222608	159,272	19.95	153,074	21.50	0.07	23.04
DB26582	Pod Mrčaru		117076	211	13.13				
DB26589	Pod Mrčaru	ERS16339501	7860184	160,331	18.68	154,540	20.30	0.09	21.35
DB26603	Pod Mrčaru	ERS16339502	11858610	188,731	25.75	181,693	29.24	0.02	31.98
DB26626	Pod Mrčaru	ERS16339503	8665574	170,472	20.52	163,913	22.43	0.04	24.38
DB26639	Pod Mrčaru		134956	269	14.90				
DB25961	Sušac	ERS16339483	11621321	189,329	25.39	182,050	28.79	0.02	31.88
DB26106	Sušac	ERS16339487	13963813	203,276	28.36	194,657	33.49	0.02	38.09
DB26355	Sušac	ERS16339489	6966010	148,096	18.02	143,171	19.15	0.10	20.22
DB26376	Sušac	ERS16339490	7192643	163,116	17.46	157,658	19.08	0.07	20.38
DB26381	Sušac	ERS16339491	8952779	176,649	20.18	169,337	22.57	0.05	24.49
DB26394	Sušac	ERS16339492	11277875	188,704	24.77	181,220	28.19	0.02	31.30
DB26395	Sušac	ERS16339493	6231584	151,887	15.96	146,854	17.32	0.12	18.28
DB26399	Sušac		1367872	32,388	9.21				
DB26437	Sušac	ERS16339495	6957250	163,081	16.67	157,485	17.93	0.06	19.01
DB26440	Sušac		180420	1,044	10.91				

Supplementary Figures

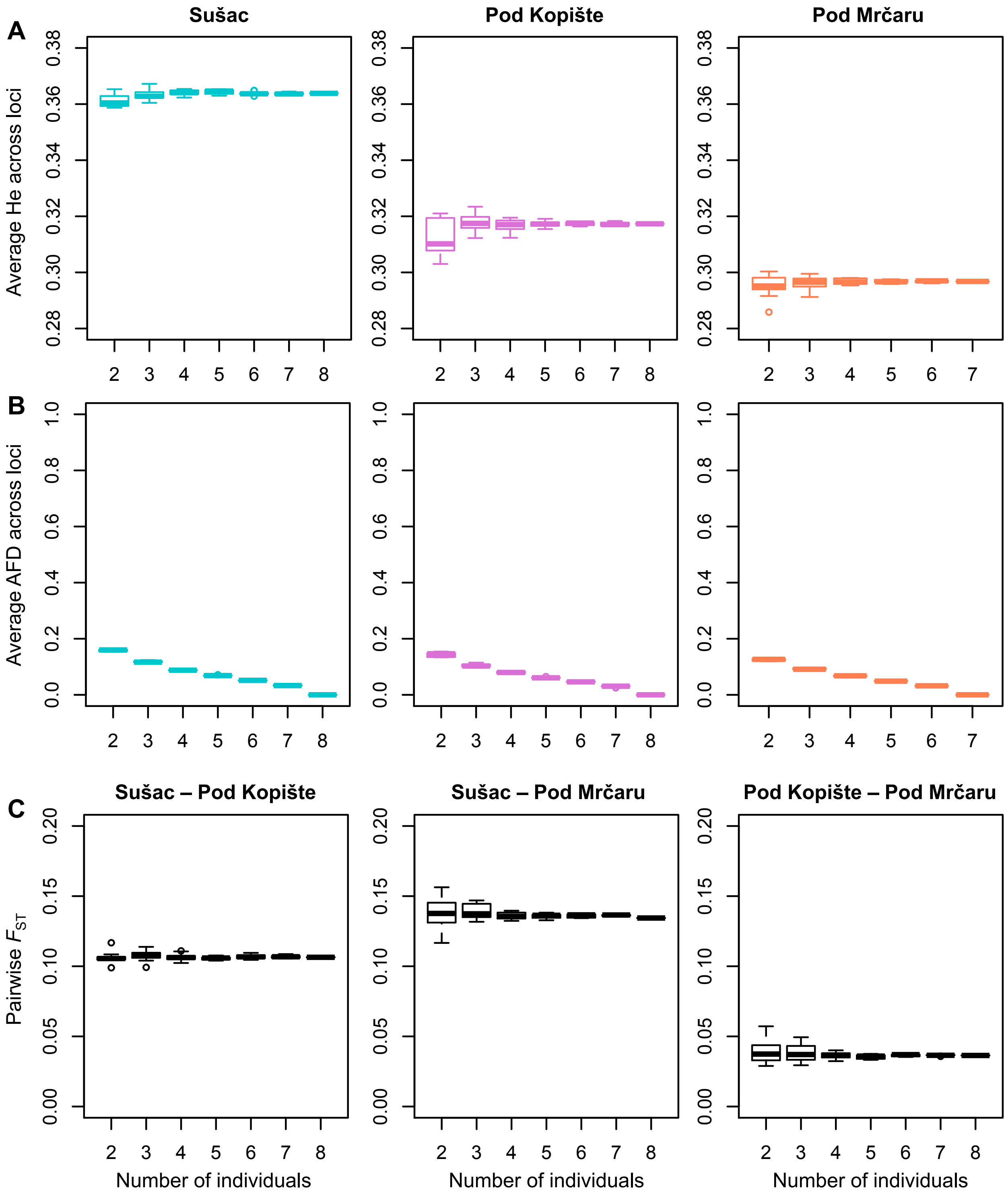

Figure S1 Evaluation of the impact of low sample sizes on the estimation of population diversity and differentiation. For each population, the analysis included 10 random subsamples among individuals. Boxplots showing the estimated A) population expected heterozygosity (He) at each locus (average across loci), B) allele frequency deviation (AFD) of AF estimated for each subsample from AF estimated for true sample size at each locus (average across loci), and C) population differentiation (Weir and Cockerham pairwise F_{ST}).

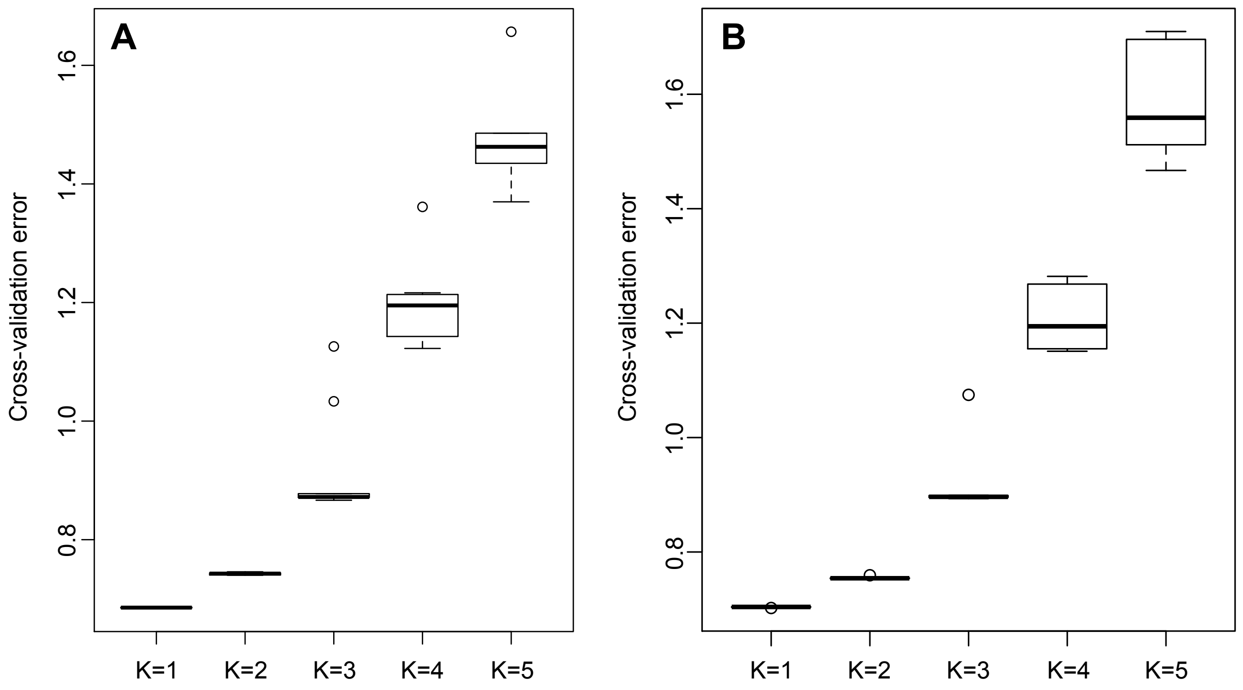

Figure S2 Determination of the optimal number of genetic clusters (K). ADMIXTURE ten-fold cross-validation error rates for K = 1 to K = 5 and 10 independent runs, showing the least error rate obtained for K = 1 for A) the 24 individuals genotyped at 54,050 SNPs including outlier loci and B) the 23 individuals genotyped at 52,470 SNPs after removing outlier loci and the admixed individual.

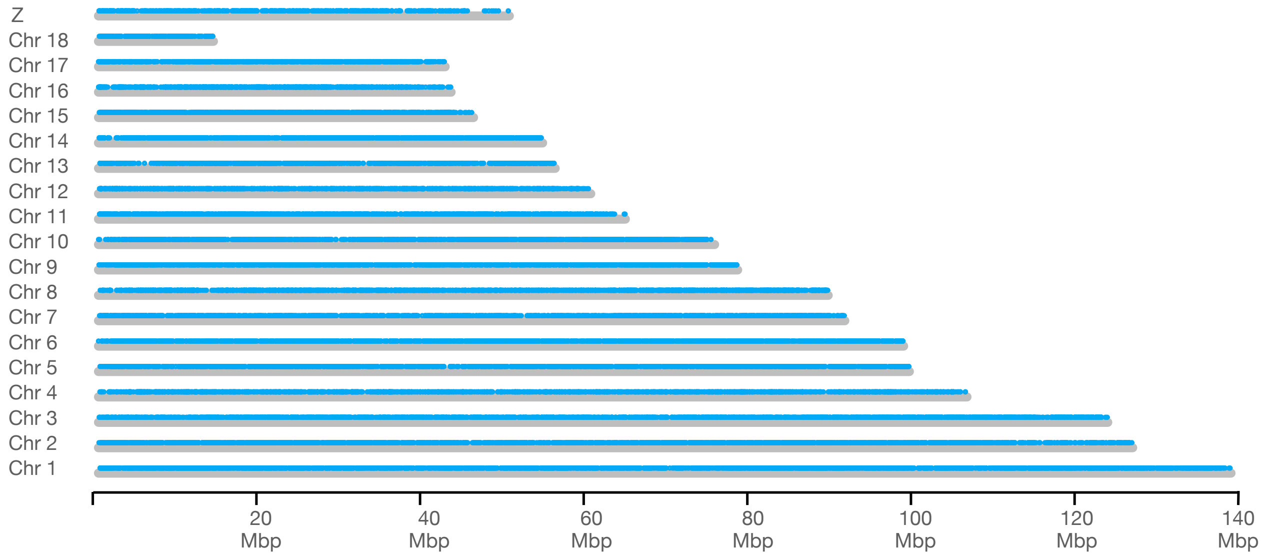

Figure S3 Schematic showing the positional information of the 52,499 *P. siculus* ddRAD loci (blue dots) aligned to the *P. muralis* reference genome (grey bars).
